# Genome-wide CRISPR/Cas9 screening identifies key profibrotic regulators of TGF-β1-induced epithelial-mesenchymal transformation and pulmonary fibrosis

**DOI:** 10.3389/fmolb.2025.1507163

**Published:** 2025-02-17

**Authors:** Chunjiang Tan, Juan Wang, Xiangrong Ye, Kaidirina Kasimu, Ye Li, Feng Luo, Hui Yi, Yifeng Luo

**Affiliations:** ^1^ Division of Pulmonary and Critical Care Medicine, The First Affiliated Hospital of Sun Yat-sen University, Guangzhou, China; ^2^ Institute of Respiratory Diseases, Sun Yat-sen University, Guangzhou, China; ^3^ Department of Emergency Medicine, The First Affiliated Hospital of Sun Yat-sen University, Guangzhou, China

**Keywords:** idiopathic pulmonary fibrosis, epithelial-mesenchymal transformation, extracellular matrix, genome-wide CRISPR/Cas9 knockout screening, TGF-β1, *COL20A1*, *COL27A1*, *WNT11*

## Abstract

**Background:**

The idiopathic pulmonary fibrosis (IPF) is a progressive and lethal interstitial lung disease with high morbidity and mortality. IPF is characterized by excessive extracellular matrix accumulation (ECM) and epithelial-mesenchymal transformation (EMT). To date, few anti-fibrotic therapeutics are available to reverse the progression of pulmonary fibrosis, and it is important to explore new profibrotic molecular regulators mediating EMT and pulmonary fibrosis.

**Methods:**

Based on our model of TGF-β1-induced EMT in BEAS-2B cells, we performed the genome-wide CRISPR/Cas9 knockout (GeCKO) screening technique, pathway and functional enrichment analysis, loss-of-function experiment, as well as other experimental techniques to comprehensively investigate profibrotic regulators contributing to EMT and the pathogenesis of pulmonary fibrosis.

**Results:**

Utilizing the GeCKO library screening, we identified 76 top molecular regulators. Ten candidate genes were subsequently confirmed by integrating the high-throughput data with findings from pathway and functional enrichment analysis. Among the candidate genes, knockout of *COL20A1* and *COL27A1* led to decreased mRNA expression of ECM components (Fibronectin and Collagen-I), as well as an increased rate of cell apoptosis. The mRNA expression of Collagen-I, together with the cell viability and migration, were inhibited when knocking out the *WNT11*. In addition, a decrease in the protein deposition of ECM components was observed by suppressing the expression of *COL20A1*, *COL27A1*, and *WNT11*.

**Conclusion:**

Our study demonstrates that the *COL20A1*, *COL27A1*, and *WNT11* serve as key profibrotic regulators of EMT. Gaining understanding and insights into these key profibrotic regulators of EMT paves the way for the discovery of new therapeutic targets against the onset and progression of IPF.

## 1 Introduction

Idiopathic pulmonary fibrosis (IPF) is an irreversible and progressive interstitial lung disease characterized by dyspnea and decline in pulmonary function (Onishchenko et al., 2022; Raghu et al., 2022). The annual incidence of acute exacerbation of IPF is high, varying from 4.8% to 43% (Collard et al., 2016). Patients with IPF experience poor prognosis, with survival rates of 88% at 1–2 years and only 31% at over 5 years (Khor et al., 2020). Of note, the median survival time for IPF patients ranges from only 2–3 years after diagnosis (Ley et al., 2011). Although the use of a few drugs (e.g., nintedanib) slowed the annual rate of decline in pulmonary function in patients with IPF (Antoniou et al., 2020), currently there are no effective therapeutics available to reverse the disease progression.

It has been demonstrated that differentiation of fibroblasts into myofibroblasts and excessive accumulation of extracellular matrix (ECM) in lung play a key role in the pathogenesis of IPF (Upagupta et al., 2018; Alvarez-Palomo et al., 2020). Importantly, prior studies have provided evidence that alveolar epithelial cells are progenitors of fibroblasts and myofibroblasts (Kim et al., 2006; Willis et al., 2006), and this conversion of alveolar epithelial cells into the mesenchymal phenotype is termed the epithelial-mesenchymal transformation (EMT). The EMT is closely related to the development of IPF. For example, there is a profound association between the EMT and transforming growth factor-beta 1 (TGF-β1) signaling pathway (Di Gregorio et al., 2020),which has been demonstrated to play a central role in pathogenesis of IPF (Ye and Hu, 2021). Although the TGF-β1 signaling has been proven as a predictive marker and pharmacological target for IPF, few anti-fibrotic drugs targeting this signaling are put into clinical use due to side effects and the need for large doses (Shi et al., 2022). Thus, the identification of new molecules that regulate the EMT and development of pulmonary fibrosis using novel high-throughput screening techniques may offer promising strategies for discovering new therapeutic targets against IPF.

In the past decade, high-throughput screening techniques, such as small interfering RNA screening (Oh et al., 2018) and microarray (Hu et al., 2018) have been applied to identify new molecular regulators contributing to the development of IPF. Despite that some genes and signaling pathways associated with IPF have been discovered, these findings vary due to specific limitations of the technology employed, as well as differences in experimental conditions and the phenotypic readout chosen for analyses. Among the high-throughput screening techniques, the genome-wide CRISPR/Cas9 screening is an advanced and powerful tool for the screening and discovery of unknown genes that contribute to specific biological phenotypes and diseases (Shalem et al., 2015). This technique has been applied to various diseases (Xu et al., 2023; Pfeifer et al., 2024) and also used for screening biological factors regulating disease susceptibility (Zhu et al., 2021). In this study, to systematically investigate molecular regulators that are closely involved in EMT and the pathogenesis of pulmonary fibrosis, we applied the genome-wide CRISPR/Cas9 knockout (GeCKO) screening technique in TGF-β1-treated human bronchial epithelial cells (BEAS-2B). Our results demonstrate that the *COL20A1*, *COL27A1*, and *WNT11* are key profibrotic regulators of IPF, and the knockout of these genes attenuated the TGF-β1-induced EMT and pulmonary fibrosis *in vitro*. Our findings show that these profibrotic regulators may serve as specific therapeutic targets, paving the way for the discovery of new medications against the onset and progression of IPF.

## 2 Materials and methods

### 2.1 Cell culture

Human bronchial epithelial cell line (BEAS-2B, CRL-3588) was purchased from the American Type Culture Collection, and 293 TN cells (LV900A-1) were obtained from the System Bioscience. Cells were cultured in Dulbecco’s Modified Eagle Medium (DMEM, Gibco, United States) supplemented with 10% fetal bovine serum (Gibco, United States). All cells were routinely tested and free from microbial contamination.

### 2.2 EMT induction by TGF-β1

Cells were seeded at a density of 1 × 10^5^ per well onto six-well culture plates and maintained in the DMEM in a humidified atmosphere of 5% CO_2_ at 37°C. On the following day, BEAS-2B cells were treated with human recombinant TGF-β1 (2 μg/mL, rTGF-β1, PHG9204, Gibco, United States) at the concentration of 5 ng/mL, 10 ng/mL, 100 ng/mL, and 1,000 ng/mL for 48 h, respectively. Cells cultured in the absence of rTGF-β1 served as the control. After the treatment, cell extracts were collected for further analyses.

### 2.3 Lentivirus production

A human genome-wide CRISPR knockout pooled library (GeCKO v2) targeting 19,050 genes and 1,864 microRNAs (miRNAs) was purchased from the Addgene (#1000000048, United States). To produce the lentivirus for the GeCKO v2 sgRNA library, 3 × 10^6^ 293 TN cells were transduced with 9 μg of GeCKO v2 plasmids, 3 μg of pCMV-VSV-G, 3 μg of pMDLg, 3 μg of pRRE, and 3 μg of pRSV-Rev in a 150 mm dish containing 90 μL polyethylenimine. After 6 h, cells were washed with PBS, and fresh complete DMEM was added. The lentiviral supernatant was harvested after 48 h of incubation at 37°C and 5% CO_2_. The supernatant was then centrifuged at 3,000 rpm for 15 min at 4°C and stored for further use.

### 2.4 Genome-wide CRISPR/Cas9 knockout library screening

Genome-Cas9-edited BEAS-2B (BEAS-2B-GeCKO) cells were generated as previously described (Wei et al., 2019). Briefly, BEAS-2B cells were transduced with GeCKO v2 sgRNA library at a low MOI (∼0.3). After incubating with puromycin for 14 days to eliminate untransduced cells, viable cells were collected to evaluate the transduction efficiency. (With regard to the knockout of individual candidate genes, corresponding gRNAs were introduced into the lentiCRISPR v2.1 vector (Supplementary Table S1), followed by the cell transduction.) Thereafter, 2 × 10^7^ BEAS-2B-GeCKO cells were treated with rTGF-β1 (10 ng/mL). We performed four consecutive rounds of TGF-β1 selection according to the EMT readout assay. After four rounds of rTGF-β1 treatment, viable cells were collected for subsequent analyses. Genomic DNA was extracted from TGF-β1-resistant BEAS-2B-GeCKO cells using the HiPure Tissue DNA Kit (Magen, China). The single guide RNA (sgRNA) sequences were amplified by polymerase chain reaction (PCR). The sequence of forward primer is 5′-TCTTGTGGAAAGGACGAAACACCG-3’; The sequence of reverse primer is 5′-ACCTTCTCTAGGCACCGGAT-3’. The gRNA sequences were amplified using 2 × PrimStar MasterMix (Takara, Japan) through the PCR. The conditions of PCR were as follows: 98°C for 30 s; 35 cycles (98°C for 10 s; 60°C for 10 s; 72°C for 50 s); 72°C for 1 min; 4°C. The distribution of sgRNA was assessed by Illumina next-generation sequencing (NGS).

### 2.5 Screening enrichment analysis

Following the NGS, the top-scoring gene hits were identified by applying a threshold of total reads of sgRNAs>300 and the sgRNA diversity (the counts of sgRNAs targeting each gene) > 1. In addition, a dataset (GSE104908) was downloaded from the Gene Expression Omnibus database and differentially expressed genes (LogFC>2, q < 0.05) were chosen for analyses. A Venn diagram was constructed using the R software v4.3.3 to compare the overlap between the top-scoring genes and GSE104908. To investigate the expression levels of the identified key profibrotic regulators in patients with pulmonary fibrosis, the GSE40839 and GSE24206 datasets were also downloaded from the Gene Expression Omnibus database. Prior to the statistical analysis, the gene chip data were subjected to deduplication, log2 transformation, and quality control. The Mann-Whitney *U* Test was applied to analyze the expression levels of key profibrotic regulators between the pulmonary fibrosis patients and control groups. Data were visualized in violin plots using the R software v4.3.3. To identify significantly enriched signaling pathways, Kyoto Encyclopedia of Genes and Genomes (KEGG) analysis were conducted using the R software v4.3.3. To characterize the biological process, cellular component, and molecular function, the Gene ontology (GO) analysis was performed using the WEB-based Gene Set Analysis Toolkit (https://www.webgestalt.org/). The STRING was used to visualize the protein-protein interaction (PPI) network.

### 2.6 RNA isolation and reverse transcription-quantitative polymerase chain reaction (RT-qPCR)

First, the 1 mL of Trizol was added to cells (5 × 10^6^) and vortexed briefly. Then 200 μL of chloroform was added to the cell lysate, followed by incubation (5 min at room temperature) and centrifugation (10,000 rpm, 5 min at room temperature). After centrifugation, 500 μL isopropanol was added to the aqueous phase and mixed gently. Samples were then centrifuged, and the supernatant was discarded. The pellet was subsequently washed with 70% ethanol, and the supernatant was removed after centrifugation. The pellet was allowed to air dry and resuspended in 20 μL of RNase-free water. The purity and concentration of RNA was measured using the NanoDrop 2000 (Thermo Fisher Scientific; United States). The cDNA was reverse transcribed using a High-Capacity cDNA Reverse Transcription Kit (Thermo Fisher Scientific; United States) according to the manufacturer’s instructions. The RT-qPCR was carried out using the PowerUp SYBR Green master mix (Applied Biosystems; United States) and target-specific primers (shown below) on a CFX96 Touch Real-Time PCR Detection System (BIO-RAD; United States). The β-actin was used as the reference gene for normalization. Relative mRNA expression of the targets was calculated as the fold change relative to PBS-treated cells using the delta-delta Ct method (2^−ΔΔCT^). Sequences of primers for target genes are listed below:

β-actin FW: TGGCACCAGCACAATGAA.

β-actin RV: CTAAGTCATAGTCCGCCTAGAAGC.

Fibronectin FW: AGCAAGCCCGGTTGTTATGA.

Fibronectin RV: CCCACTCGGTAAGTGTTCCC.

Collagen-I FW: ATTCCAGTTCGAGTATGGCGG.

Collagen-I RV: CTTGAGGTTGCCAGTCTGCT.

### 2.7 Cell transduction and Western blotting

The pLKO.1-shRNA constructs targeting *COL20A1*, *COL27A1*, and *WNT11* were purchased from IGEbio (China). The stable knockdown of these three genes was conducted through the lentiviral transduction of shRNA expressing virus, followed by puromycin selection. The pLKO.1-scramble shRNA was used as the control. The sequences of shRNAs are the following:

shRNA-*COL20A1*: GCTACACCTTGCAGATCTTCG.

shRNA-*COL27A1*: GCAGTGGCTATTCGATCTTCC.

shRNA-*WNT11*: GCCTGTGAAGGACTCGGAACT.

shRNA-scrambled: CCTAAGGTTAAGTCGCCCTCG.

After the rTGF-β1 treatment, cells were washed twice with ice-cold PBS. Cells were then lysed by adding radioimmunoprecipitation assay buffer (Beyotime, China) with the protease inhibitor (Beyotime, China), followed by centrifugation (12,000 rpm, 15 min at 4°C). After discarding the supernatant, the loading buffer (EpiZyme, United States) was added, and cells were heated at 95°C for 10 min. Samples were electrophoresed in the 10% Sodium Dodecyl Sulfate polyacrylamide gels, and proteins were subsequently transferred to polyvinylidene difluoride membranes. Membranes were blocked with skimmed milk for 2 h at room temperature and incubated with primary antibodies overnight at 4°C. On the next day, polyvinylidene difluoride membranes were washed with TBST (100 mM NaCl, 10 mM Tris, 0.1% Tween 20), and blots were incubated with horseradish peroxidase-conjugated secondary antibodies for 2 h at room temperature. After washing the TBST, membranes were developed with ECL chemiluminescent substrate (Tanon, China). Data quantification was conducted using the Fiji software (ImageJ, United States). Relative protein levels were calculated after normalization to GAPDH.

Antibodies used are as follows: rabbit anti-Fibronectin (proteintech #15613-1-AP, 1:2000), mouse anti-Collagen Type I (proteintech #67288-1-Ig, 1:5000), and mouse anti-GAPDH (proteintech #60004-1-Ig, 1:10,000). HRP-conjugated secondary antibodies include goat anti-mouse (proteintech #RGAM001, 1:5000) and goat anti-rabbit (proteintech # RGAR001, 1:5000).

### 2.8 CCK-8 assay

The cell viability was assessed using the cell counting kit-8 (CCK-8, Beyotime, China). Cells (3 × 10^3^ per well) were seeded onto 96-well plates in triplicate and incubated at 37% with 5% CO_2_ for 24 h. Cells were then treated with 10 ng/mL of rTGF-β1 for 48 h. Subsequently, 10 μL of CCK-8 solution was added, followed by an additional 2 h of incubation. The absorbance at 450 nm was detected using a microplate reader (BioTeke, United States).

### 2.9 Cell migration assay

Cells (1 × 10^4^) were seeded onto the transwell inserts (Corning, United States) containing 200 μL of serum-free DMEM. The DMEM containing 20% fetal bovine serum (500 μL) was added into the lower chamber. After 48 h of rTGF-β1 treatment (10 ng/mL), cells that migrated/invaded into the lower surface were washed with sterile PBS, fixed with 4% paraformaldehyde (20 min), and stained with 0.1% crystal violet (15 min). Excess crystal violet and unfixed cells were then washed away with PBS, followed by the observation under a bright-field light microscopy (Olympus, Japan) at ×100 magnification. Relative cell migration was calculated as the ratio of migrated cells in experimental groups to the background migration in the control group.

### 2.10 Cell apoptosis analysis

Cells (1 × 10^5^ per well) were seeded onto six-well culture plates and cultured overnight. Cells were then treated with rTGF-β1 (10 ng/mL) for 48 h. Subsequently, cells were stained with 5 μL of Annexin V-APC (BioLegend, United States) and 10 μL of 7-amino-actinomycin D (eBioscience, United States) for 15 min at room temperature. The apoptotic cells were determined and quantified by flow cytometry, and data were analyzed using the FlowJo software (Tree Star, United States).

### 2.11 Statistical analysis

All data were expressed as mean ± SEM. Data analysis was performed using the GraphPad Prism 9.30 (GraphPad Software, United States). Data were analyzed using unpaired two-tailed t-test or Mann-Whitney *U* Test for comparison between two groups. The one-way analysis of variance (ANOVA) with Tukey’s post-test was applied for multiple comparisons. The *p* value < 0.05 was considered statistically significant.

## 3 Results

### 3.1 Identification of EMT and ECM-generating phenotype in BEAS-2B and BEAS-2B-GeCKO cells

**FIGURE 1:**
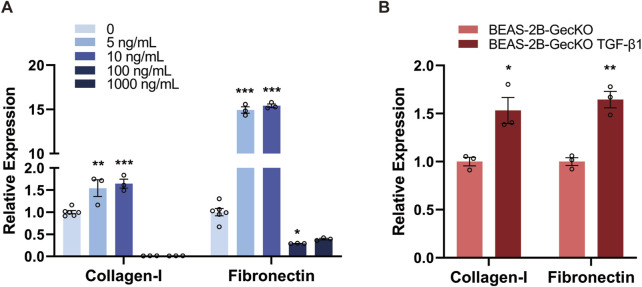
TGF-β1 treatment induces the EMT and ECM-generating phenotype in BEAS-2B and BEAS-2B-GeCKO cells. **(A)** The mRNA expression of Collagen-I and Fibronectin in BEAS-2B cells was determined by RT-qPCR following treatment with different doses of rTGF-β1. The optimized concentration of rTGF-β1 (10 ng/mL) was confirmed for subsequent experiments. **(B)** The mRNA expression of Collagen-I and Fibronectin in BEAS-2B-GeCKO cells was determined by RT-qPCR following rTGF-β1 treatment. Data are expressed as mean ± SEM, and analyzed using one-way ANOVA with Tukey’s multiple comparisons test **(A)** or unpaired two-tailed t-test **(B)**. In **(A)**, n = 6 for the control group; n = 3 for rTGF-β1 treated groups; In **(B)**, n = 3 per group. * indicates significant difference compared with the control. *P < 0.05, **P < 0.01, ***P < 0.001. *GeCKO*, Genome-wide CRISPR/Cas9 knockout; *TGF-β1*, transforming growth factor-beta 1.

To explore the successful induction of EMT in human bronchial epithelial cells and its potential association with ECM synthesis and deposition, we aimed to establish a TGF-β1-induced EMT in BEAS-2B cells and measured the level of Collagen-I and Fibronectin, both of which are essential components of ECM (Zhang et al., 2018). To identify the optimal concentration of rTGF-β1, BEAS-2B cells were treated with different doses of rTGF-β1 (0, 5, 10, 100, and 1,000 ng/mL) for 48 h, followed by detecting the mRNA expression of Collagen-I and Fibronectin. As shown in Figure 1A, the mRNA expression levels of Collagen-I and Fibronectin gradually increased with the rise of rTGF-β1 concentration, peaking at 10 ng/mL. This result demonstrates the presence of an ECM-generating phenotype in BEAS-2B cells and the successful induction of EMT. However, a significant decrease in the expression of Collagen-I and Fibronectin was observed at 100 ng/mL and 1,000 ng/mL (Figure 1A), possibly due to the detrimental effect of higher TGF-β1 concentrations on cell viability. We therefore confirmed the use of 10 ng/mL of rTGF-β1 for subsequent experiments. After four consecutive rounds of rTGF-β1 treatment, higher levels of Collagen-I and Fibronectin mRNA expression were found in BEAS-2B-GeCKO cells compared with control. This result confirms the successful induction of EMT in BEAS-2B-GeCKO cells, paving the way for the establishment of the GeCKO screening (Figure 1B).

### 3.2 Conditional screening of BEAS-2B-GeCKO cells and identification of top molecular regulators mediating EMT and pulmonary fibrosis

**FIGURE 2:**
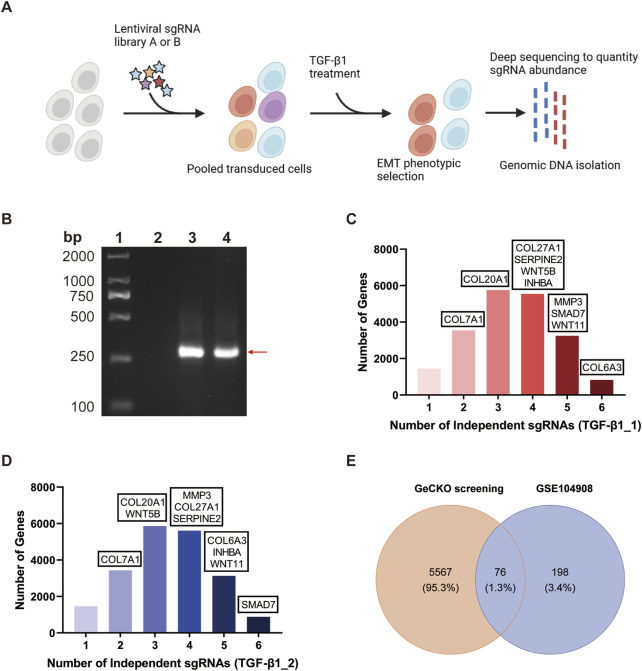
Conditional screening of BEAS-2B-GeCKO cells identifies 76 top molecular regulators mediating EMT and pulmonary fibrosis. **(A)** Schematic flow of generating BEAS-2B-GeCKO cells and subsequent TGF-β1 treatment and NGS. **(B)** A representative image demonstrating the successful transduction of GeCKO v2 sgRNA library into BEAS-2B cells. From the left to right, strips represent the markers, and the detection of GeCKO v2 sgRNA library in BEAS-2B cells, BEAS-2B-NC cells, and BEAS-2B-GeCKO cells, respectively. **(C, D)** The number of enriched genes represented by 1, 2, 3, 4, 5, or six independent sgRNAs. Genes were identified by total reads of sgRNAs and sgRNA diversity. **(E)** The Venn diagram analysis of data from our GeCKO screening and GSE104908 confirmed 76 top molecular regulators. *EMT*, epithelial mesenchymal transformation; *GeCKO*, Genome-wide CRISPR/Cas9 knockout; *sgRNA*, single guide RNA; *TGF-β1*, transforming growth factor-beta 1.

To uncover new molecular regulators of the TGF-β1-induced EMT, BEAS-2B cells were transduced with GeCKO v2 sgRNA library to generate the BEAS-2B-GeCKO cells. The Figure 2A illustrates the schematic flow of EMT induction by treating BEAS-2B-GeCKO cells with rTGF-β1 and subsequent high-throughput sequencing. We analyzed two samples in the testing set using the NGS. For each sample, a minimum of 5.1 GB of data were generated. A total of 19,050 genes and 1,864 miRNAs were detected through the NGS, in line with the number of genes and miRNAs targeted by the GeCKO pooled library. Amplified sgRNA fragments were able to be detected in BEAS-2B-NC cells and BEAS-2B-GeCKO cells as compared to BEAS-2B cells, proving that the GeCKO v2 sgRNA library was successfully transduced into BEAS-2B cells (Figure 2B). By analyzing 14M Illumina NGS reads from 3.6×10^7^ BEAS-2B-GeCKO cells post the rTGF-β1 selection, we observed the enrichment of 70,255 sgRNAs, representing 57% of total 123,411 sgRNAs in the GeCKO library. In addition, we detected an enrichment of 20,368 genes, including miRNAs, with 828 or 886 genes represented by six independent sgRNAs in the library, 3,247 or 3,132 genes represented by five independent sgRNAs, 5,553 or 5,607 genes represented by four independent sgRNAs, 5,755 or 5,861 genes represented by three independent sgRNAs, and 3,546 or 3,436 genes represented by two independent sgRNAs (Figures 2C, D).

Among the enriched genes, we identified a total of 5,643 top-scoring genes by applying the threshold of total reads of sgRNAs>300 and sgRNA diversity>1. This result suggests the potential profibrotic properties of these top-scoring genes and demonstrates that our GeCKO screening can accurately screen essential and functional molecular regulators involved in the TGF-β1-induced EMT. To identify crucial molecular regulators for further study, we compared our GeCKO screening data with the differentially expressed genes from GSE104908 (Figure 2E). Through the analysis of Venn diagram, 76 top molecular regulators were confirmed (Figure 2E).

### 3.3 Pathway and functional enrichment analysis

**FIGURE 3:**
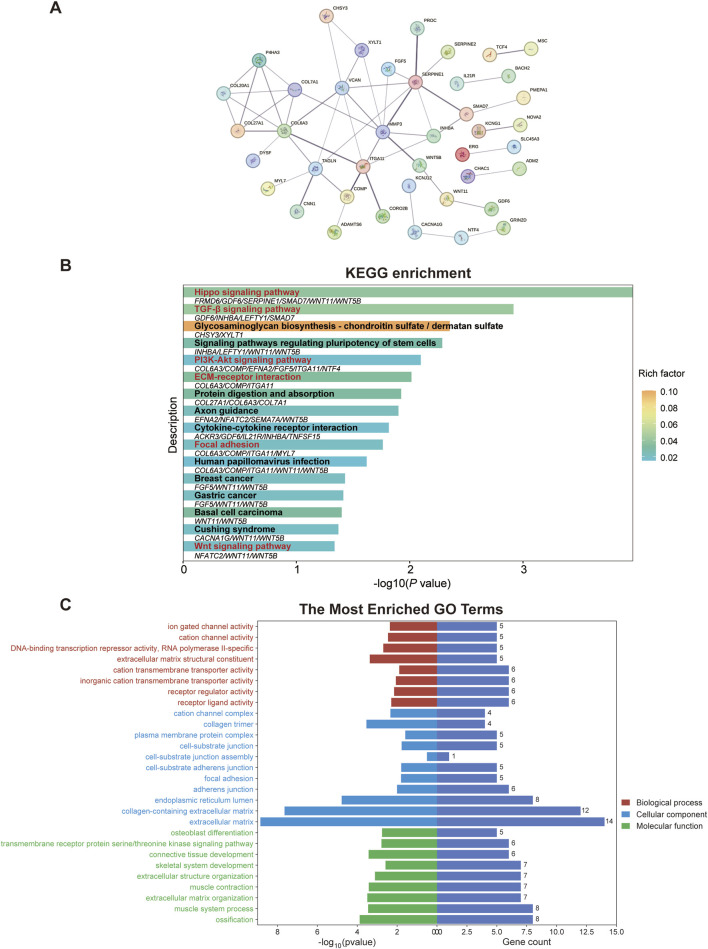
Pathway and functional enrichment analysis of top molecular regulators reveals a strong association with EMT and ECM dysregulation. **(A)** Protein-protein interaction network among the top molecular regulators was constructed in the STRING database. **(B)** KEGG pathway analysis of the top molecular regulators. Signaling pathways that are closely associated with ECM dysregulation, EMT, and pulmonary fibrosis are highlighted in red. Genes enriched in corresponding pathways are shown below the bars. **(C)** Through the GO analysis of top molecular regulators, the enriched GO terms indicate the close correlation with ECM. *ECM*, extracellular matrix; *GO*, Gene ontology; *KEGG*, Kyoto Encyclopedia of Genes and Genomes; *PI3K-Akt*, phosphatidylinositol 3-kinase-protein kinase B; *TGF-β*, transforming growth factor-beta.

To explore the PPI network among the identified top molecular regulators, we conducted the STRING analysis. As illustrated in Figure 3A, the PPI network comprising 76 nodes and 53 edges was constructed by applying a threshold of *p* < 0.05 and interaction score>0.4 (Disconnected nodes were hidden). Moreover, we performed KEGG pathway analysis for the top molecular regulators, and results exhibited enrichment in the TGF-β signaling pathway as well as other pathways associated with ECM dysregulation and EMT (e.g., focal adhesion and ECM-receptor interaction) (Figure 3B). Notably, we observed the enrichment of *INHBA* and found that the *INHBA* is involved in the TGF-β signaling pathway (Figure 3B and Supplementary Table S2). This result is in line with prior studies demonstrating the crosstalk between *INHBA*, EMT initiation and TGF-β pathway activation (Yu et al., 2021). In addition, the enrichment of *COL6A3* was found, and it participates in ECM-receptor interaction, focal adhesion, and the PI3K-Akt signaling pathway (Figure 3B and Supplementary Table S2), all of which are associated with the pathogenesis of pulmonary fibrosis. The *WNT11* and Wnt signaling pathway were also found to be enriched (Supplementary Table S2). Furthermore, the top-ranked biological process, cellular component, and molecular function of those top molecular regulators were analyzed through the GO analysis. As shown in Figure 3C and Supplementary Table S3, other molecular regulators (e.g., *COL20A1*, *COL27A1*, *COL6A3*, *SMAD7*, and *WNT11*) are involved in ECM structural constituent, collagen trimer, ECM component, adherens junction, collagen-containing ECM, as well as ECM organization. (Detailed information of KEGG and GO analyses is provided in Supplementary Tables S2, S3) Altogether, as these enriched genes are closely associated with ECM accumulation and TGF-β pathway activation, we therefore deduced that they may play a central role in regulating the TGF-β1-induced EMT and pulmonary fibrosis.

### 3.4 Confirmation of the candidate genes and the effect of their knockout on TGF-β1-induced EMT

**FIGURE 4:**
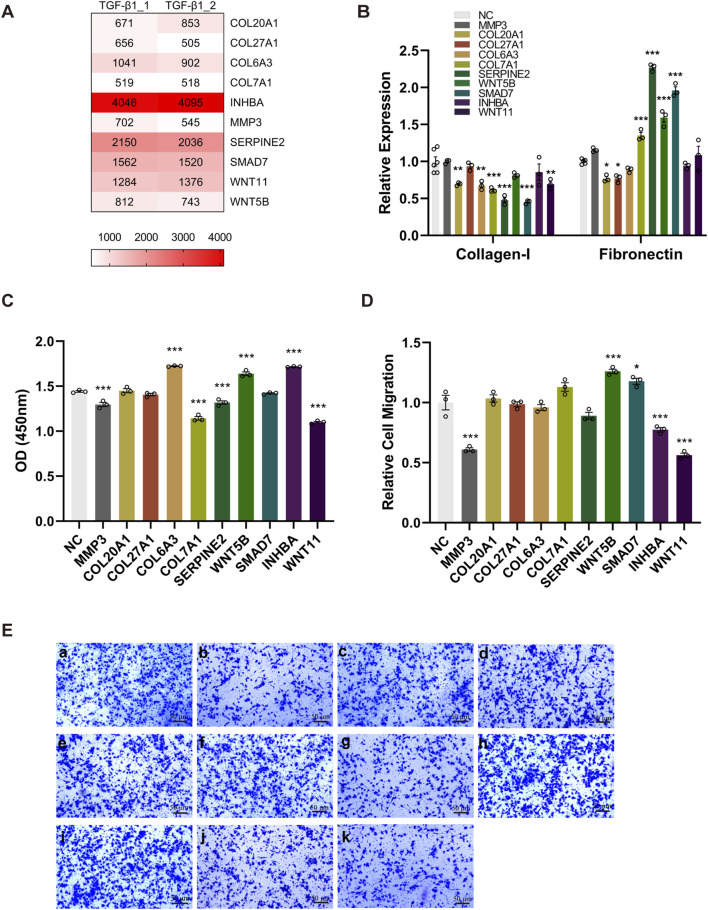
The knockout of candidate genes alters the phenotype of TGF-β1-induced EMT. Ten BEAS-2B cell lines were generated by specifically knocking out each candidate gene, followed by the rTGF-β1 treatment. PBS-treated cell lines served as the control. **(A)** A heatmap displaying the sgRNA counts of the candidate genes. **(B)** Upon the knockout of candidate genes, alterations in the mRNA expression of Collagen-I and Fibronectin was determined via RT-qPCR. **(C)** The effect of knocking out each candidate gene on cell viability was evaluated by CCK-8 assay. **(D, E)** Compared with control group (a), the effect of knocking out *MMP3* (b), *COL20A1* (c), *COL27A1* (d), *COL6A3* (e), *COL7A1* (f), *SERPINE2* (g), *WNT5B* (h), *SMAD7* (i), *INHBA* (j), and *WNT11* (k) on relative cell migration was assessed using the transwell system (magnification: ×100). Relative cell migration was calculated as the ratio of migrated cells in experimental groups to the background migration in the control group. Data are expressed as mean ± SEM, and analyzed using one-way ANOVA with Tukey’s multiple comparisons test. In **(B)**, n = 6 for the control group; n = 3 for other groups (candidate gene knockout); In **(C)** and **(D)**, n = 3 per group. * indicates significant difference compared with the control. *P < 0.05, **P < 0.01, ***P < 0.001. *NGS*, next-generation sequencing; *OD*, optical density; *sgRNA*, single guide RNA; *TGF-β1*, transforming growth factor-beta 1.

By integrating results of the Venn diagram with findings from pathway and functional enrichment analysis, the candidate genes (*COL6A3*, *MMP3*, *WNT11*, *SMAD7*, *COL27A1*, *WNT5B*, *SERPINE2*, *INHBA*, *COL20A1*, and *COL7A1*) were finally confirmed. The sgRNA counts for each candidate gene are shown in Figure 4A.

To assess whether these candidate genes play a key role in regulating the TGF-β1- induced EMT, we generated 10 BEAS-2B cell lines through the specific knockout of every single candidate gene, followed by the rTGF-β1 treatment. As presented in Figure 4B, the knockout of candidate genes (*COL20A1*, *COL6A3*, *COL7A1, SERPINE2*, *SMAD7*, and *WNT11*) greatly decreased the mRNA expression levels of Collagen-I. Moreover, although higher mRNA levels of Fibronectin were detected after knocking out the *COL7A1, SERPINE2*, *WNT5B*, and *SMAD7*, the substantial reduction in Fibronectin mRNA was observed upon the knockout of *COL20A1* and *COL27A1* (Figure 4B). In addition, post the rTGF-β1 treatment, a significant reduction in cell viability was observed when knocking out the candidate genes (*MMP3*, *COL7A1*, *SERPINE2*, and *WNT11*) compared with the control (Figure 4C). In contrast, the knockout of candidate genes (*COL6A3*, *WNT5B*, and *INHBA*) in BEAS-2B cells promoted cell proliferation (Figure 4C). Furthermore, Figures 4D, E show that the relative migration/invasion abilities of BEAS-2B cells following rTGF-β1 treatment were inhibited upon the knockout of *MMP3*, *INHBA*, and *WNT11*, whereas the knockout of *WNT5B* and *SMAD7* facilitated the migration/invasion of BEAS-2B cells.

### 3.5 The effect of knocking out key profibrotic regulators on cell apoptosis and ECM components deposition

**FIGURE 5:**
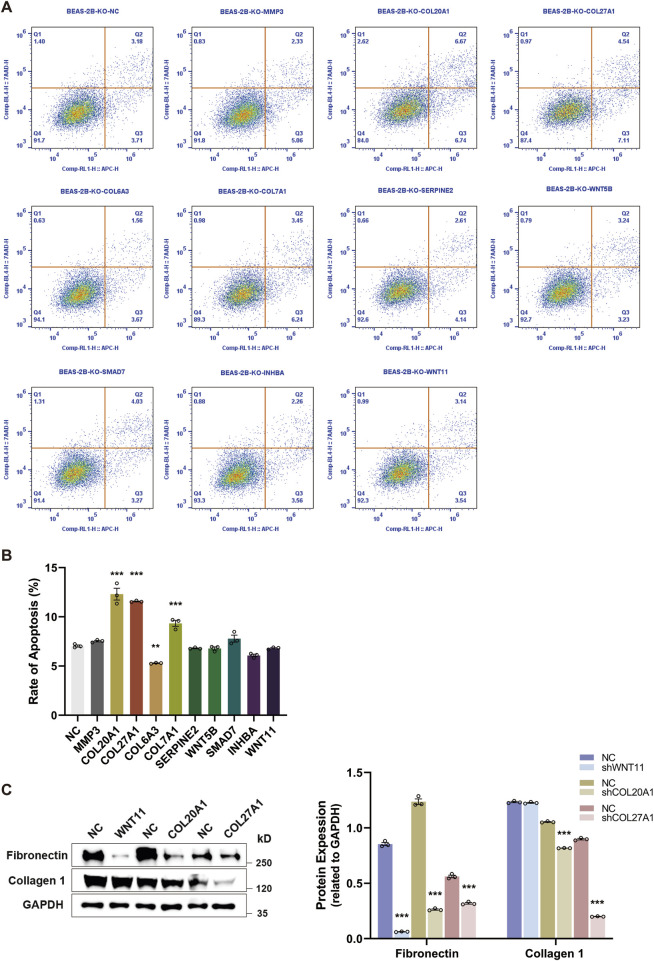
The knockout of key profibrotic regulators promotes cell apoptosis and reduces ECM components deposition. Ten BEAS-2B cell lines were generated by specifically knocking out each candidate gene, followed by the rTGF-β1 treatment. PBS-treated cell lines served as the control. **(A)** Representative plots of cell apoptosis for BEAS-2B cell lines. **(B)** Upon the knockout of candidate genes, the rate of apoptosis for BEAS-2B cell lines was analyzed by flow cytometry. **(C)** After suppressing the expression of key profibrotic regulators (*COL20A1*, *COL27A1*, and *WNT11*), alterations in the protein expression levels of Fibronectin and Collagen-I were analyzed and quantified by Western blot. Analysis of protein levels in relation to GAPDH. Data are expressed as mean ± SEM, and analyzed using one-way ANOVA with Tukey’s multiple comparisons test **(B)** or unpaired two-tailed t-test between each condition **(C)**. In **(B)** and **(C)**, n = 3 per group. * indicates significant difference compared with the control. **P < 0.01, ***P < 0.001. *KO*, knockout; *NC*, negative control.

Based on the ten BEAS-2B cell lines with specific knockout of every single candidate gene, we performed the flow cytometry to assess whether the loss-of-function of candidate genes affected the cell apoptosis. As shown in Figures 5A, B, only the knockout of *COL20A1*, *COL27A1*, and *COL7A1* greatly enhanced the rate of apoptosis in BEAS-2B cells after rTGF-β1 treatment, whereas knocking out the *COL6A3* led to a decrease in the rate of apoptosis. Moreover, after suppressing the expression of *COL20A1*, *COL27A1*, and *WNT11*, the relative protein levels of Fibronectin and Collagen-I (key fibrosis-regulating proteins) were diminished (Figure 5C). These results prove that the *COL20A1*, *COL27A1*, and *WNT11* may serve as key profibrotic regulators and contribute to the ECM deposition and TGF-β1-induced EMT.

### 3.6 The expression of *COL20A1*, *COL27A1*, and *WNT11* in patients with pulmonary fibrosis

**FIGURE 6:**
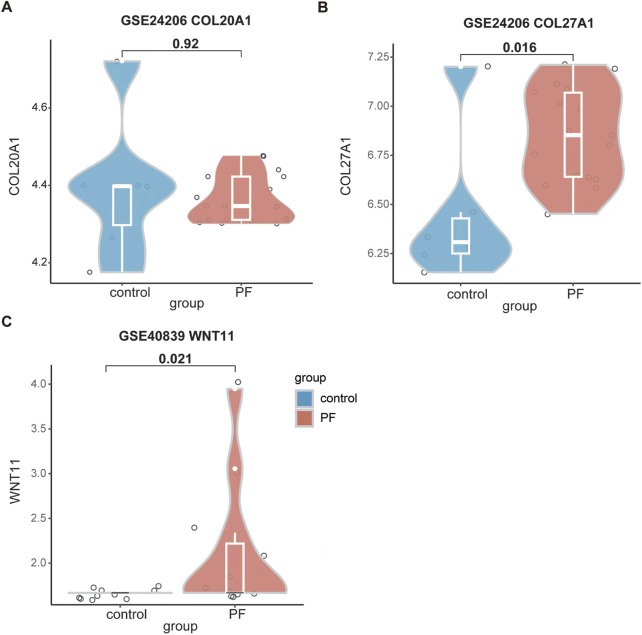
Enhanced expression levels of *COL27A1* and *WNT11* were found in patients with pulmonary fibrosis. **(A)** The expression level of *COL20A1* in GSE24206. **(B)** The expression level of *COL27A1* in GSE24206. **(C)** The expression level of *WNT11* in GSE40839. Data are shown in violin plots, and analyzed using Mann-Whitney *U* Test. In **(A)** and **(B)**, n = 6 for the control group; n = 17 for the PF group. In **(C)**, n = 10 for the control group; n = 11 for the PF group. All the violin plots were generated using R software v4.3.3. *PF,* pulmonary fibrosis.

To further consolidate the findings of our study, we analyzed the expression levels of *COL20A1*, *COL27A1*, and *WNT11* in patients with pulmonary fibrosis. As presented in Figures 6A, B, although there were no differences in the expression of *COL20A1* between IPF patients and the control in GSE24206 dataset, a substantial increase in *COL27A1* expression was observed in IPF patients. In addition, the WNT11 expression was significantly higher in patients with pulmonary fibrosis as compared with the control group in GSE40839 dataset (Figure 6C).

Altogether, all of our findings demonstrate that the *COL20A1*, *COL27A1*, and *WNT11* serve as key profibrotic regulators and may play a crucial role in regulating the TGF-β1-induced EMT and the pathogenesis of pulmonary fibrosis.

## 4 Discussion

IPF is characterized by aberrant remodeling of lung parenchyma (Li et al., 2023) that results from the chronic, continuous tissue repair process with excessive formation and deposition of collagen-rich ECM (Gauldie et al., 2002). Given that the ECM is mainly produced by fibroblasts and myofibroblasts (D'Urso and Kurniawan, 2020; Mei et al., 2021), most scholars believe that these cells are derived from resident tissue fibroblasts (Phan, 2008) and, importantly, from the epithelial cells undergoing EMT (Willis et al., 2006). Therefore, elucidating EMT-related signaling pathways and studying medications that target EMT may provide potential therapeutic strategies against the onset and progression of IPF. In order to explore the key molecular regulators of EMT and pulmonary fibrosis, the genome-wide CRISPR/Cas9 knockout technique was utilized in this research. The CRISPR/Cas9 based screening technique has proved high reagent consistency, strong phenotypic effects, and high validation rates (Shalem et al., 2015). It is a reliable and powerful method to realize genome-level analyses and to identify important genes and signaling pathways associated with the phenotype of interest, which has been used in a variety of diseases, including the organ fibrosis (Turner et al., 2020).

In the present study, we investigated the key molecular regulators of EMT by performing the GeCKO screening in TGF-β1-treated BEAS-2B cells to evaluate their phenotypic readouts, such as the abilities of cell migration/invasion and deposition of ECM components (Collagen-I and Fibronectin). By integrating the GeCKO screening results, pathway and functional enrichment analysis, and data from GSE104908, we confirmed 10 candidate genes. Among the identified candidate genes, the *COL6A3* (Nance et al., 2014), *COL7A1* (Horimasu et al., 2017), *WNT5B* (Malizia et al., 2009), and *MMP3* (DePianto et al., 2015) have been demonstrated to be associated with pulmonary fibrosis. Importantly, these aforementioned genes are involved in ECM-receptor interaction, TGF-β signaling pathway, focal adhesion, adherens junction, and Hippo signaling pathway (Figure 3). Besides the ECM-receptor interaction and TGF-β signaling pathway, the focal adhesion (Zhao et al., 2016), adherens junction (Lappi-Blanco et al., 2013), and Hippo signaling pathway (Liu et al., 2015) have also been proven to contribute to the EMT and pathogenesis of pulmonary fibrosis. Therefore, these results provide a comprehensive understanding of the critical role of these genes in the EMT and pulmonary fibrosis, paving the way for the subsequent loss-of-function experiment.

Among the confirmed candidate genes, the specific knockout of *COL20A1*, *COL27A1*, and *WNT11* in TGF-β1-treated BEAS-2B cells led to increased rate of cell apoptosis and decreased protein deposition of ECM components (Figure 5), particularly Collagen-I. As the Collagen-I plays a crucial role in fibrogenesis (Yang et al., 2013) and the pulmonary fibrosis is characterized by its accumulation (Ding et al., 2021), the reduction in Collagen-I deposition caused by knocking out the *COL20A1*, *COL27A1*, and *WNT11* may indicate a potential direction for pulmonary fibrosis treatment. Furthermore, the cell viability and migration were also inhibited when knocking out the *WNT11* (Figure 4). Importantly, significantly higher expression of *COL27A1* and *WNT11* was found in patients with pulmonary fibrosis (Figure 6). These results provide strong evidence that these three candidate genes play a key role in EMT and the pathogenesis of pulmonary fibrosis. Interestingly, enhanced expression of the collagen protein coding gene-*COL20A1* was detected in experimental models of pulmonary fibrosis (Nguyen et al., 2021; Lamichhane et al., 2022). It is noteworthy that, in contrast to these studies, we performed the loss-of-function experiment on *COL20A1* to further verify its property of promoting ECM deposition in our model of TGF-β1-treated human bronchial epithelial cells, demonstrating the crucial role of *COL20A1* in regulating the EMT and pulmonary fibrosis. Additionally, in line with our findings, a strong upregulation of *COL27A1* was also found in IPF lungs (Selman et al., 2006), indicating its potential role in mediating the onset and progression of pulmonary fibrosis. Indeed, our loss-of-function experiment further confirmed the key role of *COL27A1* in facilitating ECM deposition and TGF-β1-induced EMT because the specific knockout of *COL27A1* attenuated the deposition of ECM components. Of note, apart from its role in ECM deposition, the expression of *COL27A1* has been reported to be associated with apoptosis to maintain the tissue homeostasis (Gonzaga-Jauregui et al., 2020). Since elevated levels of cell apoptosis were observed upon the knockout of *COL27A1*, we assume that the enhanced expression of *COL27A1* may reduce cell apoptosis, thereby promoting the survival and proliferation of cells with ECM-generating phenotype and then resulting in the development of pulmonary fibrosis. Consistent with our assumptions, impaired apoptosis in myofibroblasts (Chang et al., 2010) and apoptosis-resistant fibroblasts (Ajayi et al., 2013) are found in the IPF lung. Surprisingly, blocking the inhibitor of apoptosis proteins abrogated bleomycin-induced pulmonary fibrosis (Ashley et al., 2016), suggesting that induction of apoptosis in fibrosis effector cells may serve as an alternative therapeutic strategy for the treatment of IPF. With regard to the *WNT11*, a previously published study reported a remarkable increase of *WNT11* in renal epithelial cells following TGF-β1 treatment, and the *WNT11* interacted with TGF-β for the development of renal fibrosis (Zhang et al., 2012). Despite that prior studies have investigated the role of *WNT11* and Wnt signaling in both *in vivo* and *in vitro* models of pulmonary fibrosis (Henderson et al., 2010; Liu et al., 2021), the direct molecular targets or downstream signaling of *WNT11* and the mechanism by which *WNT11* interacts with other profibrotic molecules to regulate the pathogenesis of pulmonary fibrosis still remain unclear. This requires to be further explored by future studies. Moreover, although our results show that significantly elevated expression of *COL27A1* and *WNT11* was found in pulmonary fibrosis patients, we did not find any diagnostic or prognostic information related to these genes, indicating that further *in vivo* experiments and preclinical studies require to be carried out to explore their potential diagnostic and prognostic value in pulmonary fibrosis. Notably, our results present that silencing *WNT5B* promoted the TGF-β1-induced EMT by enhancing cell viability and mRNA expression of Fibronectin, as well as the cell migration/invasion abilities. Nevertheless, the suppression of *WNT5B* mitigated the EMT in EBV-treated epithelial cells (Malizia et al., 2009). This suggests that the regulatory mechanism of some candidate genes needs to be further verified using *in vivo* models of pulmonary fibrosis. Collectively, by combining the GeCKO screening with loss-of-function experiment, our results provide a more profound and reliable validation on the profibrotic properties of the candidate genes identified in this study, and the *COL20A1*, *COL27A1*, and *WNT11* were confirmed as key profibrotic regulators.

Our study has several limitations. First, due to limited resources, we focused on screening and investigating key profibrotic regulators of pulmonary fibrosis using the GeCKO screening and loss-of-function experiment in the present study. In our follow-up experiments, the overexpression of key profibrotic regulators and multiple experimental techniques will be applied to explore potential molecular targets and downstream profibrotic pathways that these key profibrotic regulators may interact with for the pathogenesis of IPF. Second, we performed the GeCKO screening and subsequent loss-of-function experiment *in vitro*, whereas the impact of systemic and organ milieu and the potential crosstalk between different effector cells cannot be overlooked in the onset and development of IPF. Therefore, we already established the mouse model of bleomycin-induced pulmonary fibrosis, and we are planning to further investigate the profibrotic properties of the key profibrotic regulators using the *in vivo* GeCKO library screening technique in our future research.

## 5 Conclusion

Through the combination of GeCKO library screening, pathway and functional enrichment analysis, and other experimental techniques, we identified 10 candidate genes for TGF-β1-induced EMT. By applying the loss-of-function experiment, the *COL20A1*, *COL27A1*, and *WNT11* were confirmed as key profibrotic regulators of EMT and development of pulmonary fibrosis. Gaining understanding and insights into these profibrotic regulators may promote the identification of novel therapeutic targets for the prevention and treatment of IPF.

## Data Availability

The data presented in the study are deposited in the NCBI SRA repository, accession number PRJNA1221872.

## Supplementary material

SUPPLEMENTARY TABLE S1 Sequences and total reads of sgRNAs targeting the candidate genes.

SUPPLEMENTARY TABLE S2 Results of KEGG pathway analysis for the 76 top molecular regulators.

SUPPLEMENTARY TABLE S3 Results of enriched GO terms for the 76 top molecular regulators, categorized by biological process, cellular component, and molecular function.

## Abbreviations

ANOVA, analysis of variance; CCK-8, cell counting kit-8; DMEM, Dulbecco’s Modified Eagle Medium; ECM, extracellular matrix; EMT, epithelial-mesenchymal transformation; GeCKO, genome-wide CRISPR/Cas9 knockout; GO, Gene ontology; IPF, idiopathic pulmonary fibrosis; KEGG, Kyoto Encyclopedia of Genes and Genomes; miRNAs, microRNAs; NGS, next-generation sequencing; PBS, phosphate buffered saline; PCR, polymerase chain reaction; PPI, protein-protein interaction; RT-qPCR, Reverse transcription-quantitative polymerase chain reaction; sgRNA, single guide RNA; TGF-β, transforming growth factor-beta.
